# Mesenchymal Stromal Cells for Heart Diseases: Current Perspective and Future Directions

**DOI:** 10.3390/biom16101506

**Published:** 2026-10-15

**Authors:** Miłosz Andrzej Zajączkowski, Milena Deptuła-Knioła, Małgorzata Zawrzykraj-Motylińska, Agata Kaczyńska-Miotk, Michał Pikuła, Adam Kosiński

**Affiliations:** 1 Division of Clinical Anatomy, Department of Anatomy, Faculty of Medicine, Medical University of Gdansk, Dębinki 1, 80-211 Gdańsk, Poland; milosz.zajaczkowski@gumed.edu.pl (M.A.Z.); malgorzata.zawrzykraj-motylinska@gumed.edu.pl (M.Z.-M.); agata.kaczynska-miotk@gumed.edu.pl (A.K.-M.); 2 Laboratory of Tissue Engineering and Regenerative Medicine, Division of Embryology, Faculty of Medicine, Medical University of Gdansk, Dębinki 1, 80-211 Gdańsk, Poland; milena.deptula-kniola@gumed.edu.pl (M.D.-K.); michal.pikula@gumed.edu.pl (M.P.)

**Keywords:** mesenchymal stromal cells, cardiovascular diseases, cardiac regeneration, cell therapy, regenerative medicine, tissue engineering, biomaterials, cell delivery

## Abstract

Cardiovascular diseases remain the leading cause of mortality worldwide and continue to impose a substantial clinical burden despite major advances in pharmacological and interventional therapies. Because the adult myocardium has limited regenerative capacity, irreversible cardiomyocyte loss frequently leads to adverse remodeling and progressive heart failure (HF). Mesenchymal stromal cells (MSCs) are among the most extensively studied cell therapies for cardiac repair because of their immunomodulatory, pro-angiogenic, anti-inflammatory, and anti-fibrotic effects, mediated predominantly through paracrine signaling. This review summarizes the current evidence for MSC-based therapy in ischemic heart disease (IHD), coronary and microvascular disorders, myocardial infarction, HF, myocarditis, pulmonary arterial hypertension, and valvular heart disease (VHD). We discuss MSC sources, preclinical and clinical evidence, manufacturing and dosing, delivery routes, biomaterial-assisted transplantation, preconditioning, genetic modification, and imaging for cell tracking and treatment monitoring. Although MSC therapy has shown a favorable safety profile and regenerative potential, clinical efficacy remains variable because of limited cell survival, poor myocardial retention, and heterogeneous treatment protocols. Further optimization of manufacturing, delivery, and patient selection, together with well-designed randomized trials, is required to establish standardized protocols and support clinical translation.

## 1. Introduction

Cardiovascular disease (CVD) continues to be the leading cause of death worldwide, responsible for an estimated 19.2 million deaths in 2023, roughly one in three deaths globally [1]. It places a heavy burden on patients, their families, and health-care systems [2]. This burden is projected to grow further, with the global prevalence of CVD estimated to rise from 598 million in 2025 to 1.14 billion in 2050, and annual cardiovascular mortality projected to increase from 20.5 million deaths in 2025 to 35.6 million in 2050 [3].

Despite advances in pharmacotherapy and interventional cardiology, many patients still sustain irreversible myocardial injury and eventually develop chronic HF. Current therapies slow disease progression but cannot reverse myocardial damage [4,5]. Because mature cardiomyocytes have little regenerative capacity after injury, cell-based therapies have become an attractive therapeutic strategy [4].

Extensive research is currently focused on the use of MSCs in regenerative medicine, including CVD [5]. In the cardiovascular system, these cells can participate in repair by reducing inflammation and promoting differentiation of surrounding myocardial cells [6]. MSCs can also inhibit fibrosis and increase resistance to apoptosis [7].

According to the International Society for Cellular Therapy, MSCs should meet minimal criteria. These include adherence to plastic in standard conditions, specific phenotype (positive ≥ 95%) for CD105, CD90, CD73 and negative (≤2%) for CD45, CD34, CD14 or CD11b, CD79a or CD19, HLA-DR) and ability to differentiate in vitro into osteoblasts, adipocytes and chondroblasts [8,9]. MSCs are best known for their paracrine activity, which is believed to be one of their mechanisms of action. They secrete various bioactive factors, including extracellular vesicles, cytokines, chemokines, and growth factors [10]. They also communicate with neighboring cells through extracellular vesicles such as exosomes. Their secretome contains angiogenic mediators, including vascular endothelial growth factor (VEGF), insulin-like growth factors (IGF-1), and hepatocyte growth factor (HGF). For this reason, MSCs have been referred to as “medicinal signaling cells”, a term introduced by A. Caplan to emphasize that their principal therapeutic effects arise from the secretion of bioactive signaling molecules rather than from direct tissue replacement [11]. MSCs also produce extracellular matrix proteins, including collagens, fibronectin, and laminin, while expressing adhesion molecules such as integrins and cytoskeletal proteins such as vimentin [12].

Within the cardiovascular system, MSCs can exert a protective effect on the myocardium by modulating the inflammatory response and enhancing angiogenesis [13]. In addition, these cells increase the resistance of tissues to apoptosis and limit the development of fibrosis, which is why they are regarded as a potentially effective tool in regenerative therapy of the heart and blood vessels. The therapeutic function of MSCs is associated with their paracrine activity, primarily through the secretion of a range of biologically active substances into the environment in which they reside [7,14,15].

The greatest challenge of MSC-based therapies is undoubtedly the issue of technical limitations, which include low cell retention in the ischemic environment, reduced survival of the transplanted cells, and the need for further optimization of both the route of administration and the timing of transplantation [7]. Furthermore, regulatory challenges, including manufacturing compliance with good manufacturing practice (GMP), quality control, and approval requirements, continue to hinder the broad clinical translation of MSC-based therapies.

In this review, we summarize current knowledge about MSC use in the treatment of CVD. We describe sources of MSCs that are used for cardiac repair. We also describe the technical aspects of their preparation and dosing, as well as routes of administration. Furthermore, we discuss strategies for enhancing cell potential and retention, as well as methods for imaging and monitoring the transplant. Moreover, we characterize common cardiac diseases and describe clinical and preclinical applications of MSCs in their treatment.

## 2. Sources of MSCs

**Figure 1:**
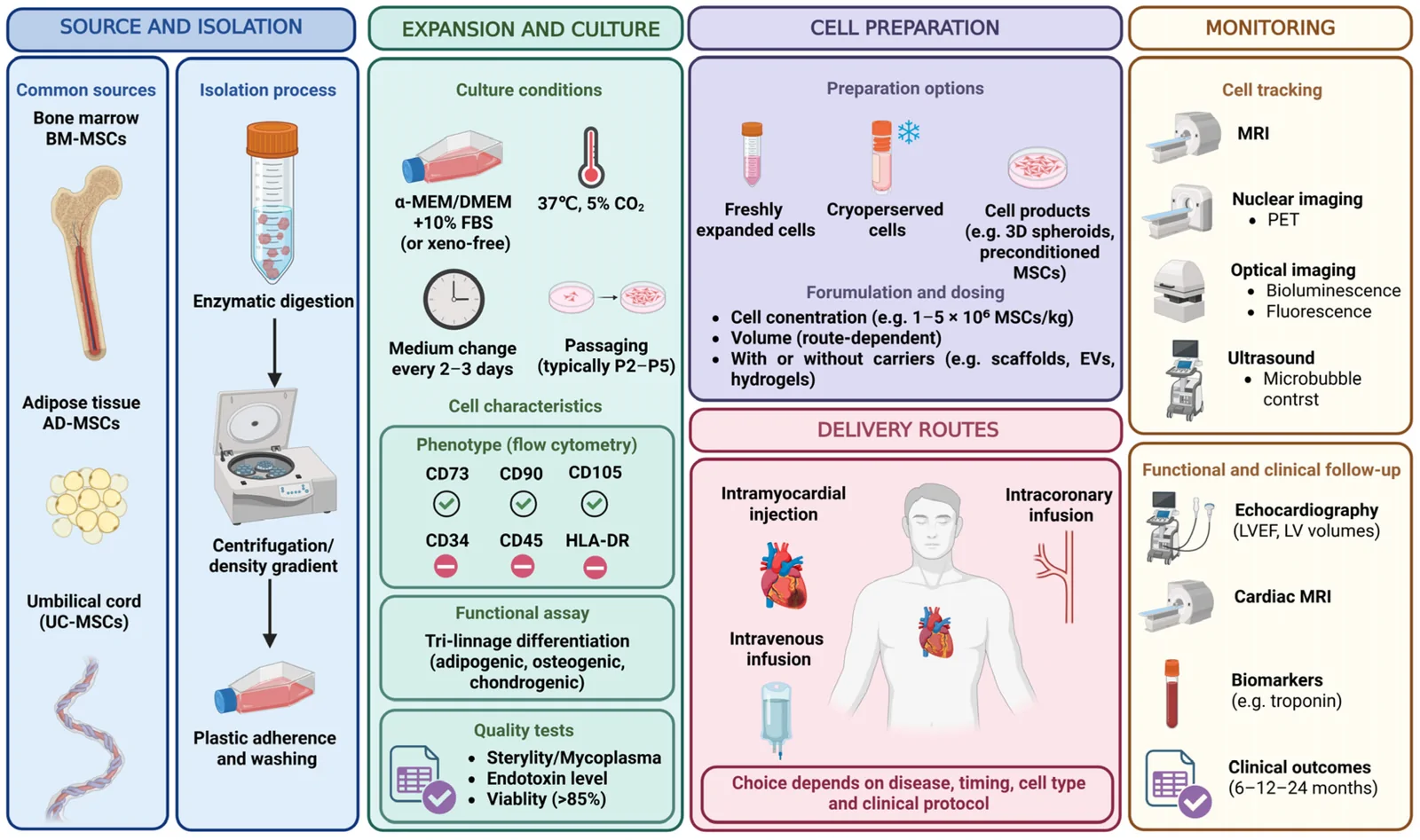
Workflow of MSC-based therapy for cardiovascular diseases, highlighting cell sourcing, expansion, preparation, delivery routes, and monitoring approaches that determine therapeutic outcomes. MSCs can be isolated from various tissue sources, including bone marrow, adipose tissue, and umbilical cord, followed by ex vivo expansion under standardized culture conditions. After quality control and phenotypic characterization, cells may be administered as freshly expanded cells, cryopreserved preparations, or engineered cell products. Therapeutic delivery can be achieved through intravenous infusion, intracoronary infusion, or intramyocardial injection. Treatment efficacy and cell fate are subsequently assessed using imaging-based cell tracking, cardiac imaging modalities, circulating biomarkers, and clinical outcome measures. Created in BioRender. Deptuła, M. (2026) https://BioRender.com/htzv8lf.

Mesenchymal stromal cells are regarded as one of the most promising tools of cell therapy. One of the fundamental features of MSCs is their potential to differentiate in vitro into three cell lineages: osteoblasts, adipocytes, and chondroblasts [8]. MSCs can be obtained from many different tissues of the body, of which the most frequently studied and used sources are cells derived from bone marrow, adipose tissue, and the umbilical cord [16]. Although some studies indicate biological differences between MSCs derived from different sources, their regenerative potential may largely depend on the study methodology, donor characteristics, and the method of cell isolation and assessment (Figure 1) [17]. Beyond methodological variations and donor characteristics, tissue origin may substantially influence MSC biological behavior, including their differentiation potential and hormonal sensitivity. For instance, recent studies have demonstrated that tissue source can influence PTH1R-dependent signaling cascades, thereby determining pro- or anti-osteogenic responses to hormonal stimuli [18]. Such origin-specific traits highlight that MSC populations from distinct tissue niches may possess specialized functional programs rather than being functionally equivalent.

In cardiac regenerative medicine, the most commonly used MSCs are those derived from bone marrow (BM-MSCs), owing to their regenerative properties, particularly in the case of myocardial infarction (MI) and HF. Bone marrow is a rich source of hematopoietic stem cells [19,20]. Obtaining MSCs from bone marrow involves an aspiration procedure, performed by puncturing and aspirating liquid marrow, usually from the iliac crest [21].

MSCs isolated from adipose tissue (AD-MSCs) are characterized primarily by their high angiogenic potential [20]. This type of MSC is readily available, as it can be obtained from the tissue in a minimally invasive manner. AD-MSCs are isolated from subcutaneous adipose tissue, which may be derived from liposuction or obtained as waste following a surgical procedure [22,23].

Both BM-MSCs and AD-MSCs share the characteristic of being easy to obtain and able to be expanded ex vivo. In addition, they exhibit low immunogenicity, which is extremely important with regard to the reduced risk of rejection by the immune system in allogeneic cell transplants [24].

UC-MSCs are stromal cells derived from umbilical cord tissue. They are isolated from various parts of the umbilical cord, including Wharton’s jelly (WJ-MSCs), umbilical cord blood (UCB), and the subendothelium of the umbilical vein [25,26]. Such cells possess a high proliferative potential and anti-inflammatory activity [27].

Furthermore, when evaluating bone marrow and adipose tissue as primary MSC sources for cardiac regeneration, the biological implications of their multilineage differentiation potential should be considered, particularly their differential propensity toward osteogenic, chondrogenic, and adipogenic lineages [28,29]. Although the therapeutic efficacy of MSCs in the heart is now recognized as predominantly paracrine rather than direct transdifferentiation, lineage-specific priming inherent to tissue origin remains clinically relevant [29,30]. Bone marrow-derived MSCs retain a strong osteo-chondrogenic propensity, raising a theoretical consideration regarding the potential for undesired osteogenic activity in the cardiac microenvironment, whereas adipose-derived MSCs exhibit a pronounced adipogenic bias [28,29]. Importantly, tissue source may also influence the composition of the MSC secretome, which is considered a major mediator of their therapeutic effects [31].

In addition to systemic adult niches such as bone marrow and adipose tissue, cardiac-resident mesenchymal stromal cells (cMSCs) have emerged as a physiologically relevant tissue-specific population. Naturally residing within the atrial and ventricular interstitium, epicardium, and perivascular spaces, cMSCs possess intrinsic cardiogenic commitment and paracrine profiles tailored to the myocardial microenvironment [32]. When compared directly with bone marrow-derived MSCs (BM-MSCs), cMSCs exhibit enhanced basal expression of cardiac-specific proteins and gap junctional channels (e.g., connexin-43), facilitating functional electrophysiological integration and paracrine cardioprotection [32]. However, the translational utility of autologous cMSCs is strictly dictated by the underlying health status and pathophysiological background of the donor patient. Recent evidence demonstrates that chronic cardiac pathologies—such as IHD, infective endocarditis, and congenital anomalies like bicuspid aortic valve (BAV) disease—induce profound disease-associated remodeling in cMSCs. cMSCs harvested from diseased hearts or young BAV patients display a prematurely aged phenotype characterized by cellular senescence, altered mitochondrial oxidative metabolism, impaired differentiation potential, and a shift in their secretome from anti-arrhythmic and cytoprotective signaling (e.g., IGF-1) toward a pro-inflammatory profile rich in IL-6 and IL-1β [33,34]. These findings indicate that donor health status should be considered when evaluating the therapeutic potential of autologous cMSCs and may support the investigation of standardized allogeneic platforms or regenerative preconditioning strategies.

MSC transplantation for the treatment of cardiac diseases can be conducted in either an autologous or an allogeneic setting. In autologous transplantation, MSCs are harvested from the patient undergoing treatment. In contrast, allogeneic MSC transplantation involves the use of cells obtained from a healthy donor. The choice between autologous and allogeneic MSC sources in regenerative cardiology is associated with specific clinical challenges and considerations [35].

Concurrently, donor-related variability represents a major determinant of autologous MSC potency [36]. Autologous cells harvested from older patients or individuals with chronic CVD, HF, metabolic syndrome, or diabetes frequently suffer from “autologous dysfunction” [37]. This disease- and age-associated impairment is characterized by premature cellular senescence, elevated reactive oxygen species (ROS) levels, reduced proliferative capacity, down-regulated CXCR4 expression (impairing homing), and an altered, pro-inflammatory secretory phenotype [38]. Such donor heterogeneity may contribute to variability in therapeutic responses in autologous settings, reinforcing the clinical shift toward screened, healthy allogeneic donors or the application of ex vivo preconditioning to rejuvenate impaired autologous MSCs [36].

In autologous transplantation, the main sources of MSCs are cells derived from bone marrow or adipose tissue [39]. The main advantages of this type of transplantation include, above all, the absence of the risk of rejection by the immune system. Unfortunately, autologous transplantation also entails certain limitations, including the fact that the quality of MSCs depends on the health status of the patient, and thus of the donor. In older cardiac patients, MSCs have a reduced capacity for regeneration and proliferation. The therapeutic potential of MSCs is also weakened as a result of other chronic diseases and the patient’s lifestyle [34,37,40]. In addition, after harvesting, MSCs require ex vivo expansion, which unfortunately precludes their use in the acute phase, e.g., after a myocardial infarction [16,41].

Both autologous and allogeneic MSCs have been extensively investigated in clinical studies. Allogeneic MSCs are particularly attractive for clinical translation because they can be obtained from healthy donors, expanded and standardized in advance, and developed as readily available “off-the-shelf” cell products [42,43,44]. Allogeneic transplants confer a range of positive effects in regenerative therapy, in particular because the cells originate from young, healthy individuals, which ensures their high viability and high potential for division. In addition, the MSCs are prepared in advance and ready for immediate administration in emergency situations. Cells obtained from a single donor can be used in many recipients, which contributes to reducing the costs of manufacturing the therapy [41,43,45]. MSCs exhibit immunological properties that enable them to be used safely in allogeneic transplants, without the need for immunosuppressive drugs. This results from the low expression of HLA-I molecules and the minimal or absent expression of HLA-II and costimulatory factors, which limits the activation of the recipient’s immune response and enables the survival of the transplanted cells in the body [46,47].

Clinical studies have shown that both types of transplants are equally safe in patients with HF [48]. In addition, some data suggest that allogeneic cells may be more effective in improving endothelial function than the patient’s own cells [49]. In an inflammatory environment, however, allogeneic MSCs may change their state from immunomodulatory to immunostimulatory, which leads to their faster clearance by the recipient’s body [44]. Regardless of the MSC source, most of the transplanted cells disappear from cardiac tissue within a short time. Therefore, one should bear in mind the paracrine effect of MSCs, which consists of the stimulation of reparative processes in tissues [50].

## 3. Technical Aspects of MSC Transplantation and Dosing in Heart Diseases

Regardless of the origin of the MSCs, a very important aspect is the isolation and culture of the cells that are to be used for transplantation. Such a process is multistage and must be subject to strict standards that ensure the reproducibility of clinical results as well as the sterility and viability of the cells. Accordingly, MSC isolation is carried out in accordance with the principles of GMP [51].

In addition to ensuring cell viability and yield, a fundamental requirement of clinical-grade MSC production under GMP is the mandatory confirmation of microbiological safety across all manufacturing stages [52,53]. Multi-tiered testing protocols must be implemented, including entry control of raw materials, in-process monitoring during culture media changes, and final product release testing prior to clinical administration. These mandatory quality controls strictly encompass tests for aerobic and anaerobic bacterial and fungal sterility, rapid or culture-based detection of Mycoplasma species, quantification of bacterial endotoxins (e.g., using the Limulus amebocyte lysate [LAL] assay), and viral screening. Adherence to these regulatory standards is essential for minimizing the risk of product contamination and ensuring the microbiological safety of MSC-based medicinal products [53,54].

In the first stage, the biological material from which the MSCs are to be isolated is collected. As mentioned earlier, cells are most often obtained from bone marrow in the form of several dozen milliliters of aspirate from the iliac bone, drawn into syringes containing heparin to prevent clotting. Cells derived from adipose tissue, most often obtained during liposuction, are also used, as are cells derived from the umbilical cord and Wharton’s jelly after delivery [51,55,56].

Subsequently, mechanical separation is performed. Solid tissues, e.g., adipose tissue or the umbilical cord, are cleaned and fragmented, and then subjected to enzymatic separation, most often using type I or type II collagenase. This process releases the cells from the extracellular matrix. At this stage, the cell suspension is centrifuged, and the stromal vascular fraction (SVF)—which contains MSCs, endothelial cells, pericytes, and other stromal cells—is separated [57,58,59].

After isolation, the MSCs are seeded into culture vessels. For this purpose, the adhesion of MSCs to plastic is exploited—that is, their unique ability to adhere to the surface of culture vessels—which allows them to be separated from non-adherent hematopoietic cells [8]. Then, after 24–48 h, the cells that have not adhered to the substrate are removed together with a medium change, so that only cells with a fibroblast-like morphology remain [55]. The preliminary suspension thus prepared is filtered through sieves with a mesh size of 40–100 μm, thereby removing tissue debris [15,60].

Because the number of MSCs in the starting material is small, it is necessary to expand them ex vivo in order to achieve a therapeutic dose. The cells are placed in incubators at a temperature of 37 °C, in an atmosphere of 5% CO_2_ and high humidity [15,61]. The cells are most often cultured in basal media such as Dulbecco’s Modified Eagle Medium (DMEM) or alpha-Minimum Essential Medium (α-MEM), supplemented with growth factors. Increasingly, in clinical studies, human platelet lysate (hPL) or human serum (of the AB group) is used as a supplement. This is intended to avoid the risk of xenogeneic infections and immune reactions [62,63]. After the cells reach an appropriate density, on average 80% confluence, they are enzymatically detached from the culture bottle using, e.g., trypsin, and then transferred to subsequent vessels for further expansion. This process is repeated over 2 to 4 passages [15,51,61].

Preparation of the therapeutic dose requires precise counting of live cells. In cardiac studies, doses ranging from several million to over 700 million cells are most often used. It is worth adding, however, that there is still no single established cell dose in clinical studies, and therefore this lack of consensus accounts for such a wide numerical range of cells used per dose. Accordingly, the cell dose is often calculated per patient body weight according to the formula: 1–5 × 10^6^ MSCs/kg [62,64].

After obtaining an appropriate number of cells, the MSCs are prepared as a cell suspension in a suitable isotonic carrier. After the final passage, the cells are washed and suspended in, e.g., 0.9% NaCl, phosphate-buffered saline (PBS), or other commercial buffer kits. Additives intended to stabilize the suspension and prevent aggregation, e.g., human albumin, may also be added to such a suspension [51]. In order to minimize the risk of embolism, the resulting suspension may be subjected once more to a filtration process to ensure safety during administration [51,65]. During the preparation of the suspension, it is extremely important to avoid vigorous shaking and rapid aspiration with a needle, because the delicate cell membranes of MSCs may thereby be mechanically damaged [51]. The cells thus prepared are administered directly to the patient or subjected to cryopreservation. MSCs can be used fresh, straight after culture, or after cryopreservation and thawing. The choice depends mainly on three factors: the type of transplantation (autologous or allogeneic), the logistics of the therapeutic process, and the time since the cardiovascular event [66].

Cryopreservation of MSCs enables their long-term storage, immediate availability for therapy, better product standardization, and the possibility of administering multiple doses. In the treatment of HF or MI, freezing MSCs enables the development of allogeneic therapies, since a ready-made MSC preparation can be administered immediately after the patient has been qualified for this type of therapy [67,68]. Cryopreserved cells are most often stored in liquid nitrogen in a solution with a cryoprotectant, e.g., dimethyl sulfoxide (DMSO). This halts their metabolism and cell division [69]. It is worth adding, however, that in the case of frozen MSCs, a partial loss of viability, a reduction in immunomodulatory activity, and a transient decrease in proliferation may occur [70,71]. In order to use frozen cells, MSC protocols often recommend, after thawing, refreshing them by washing out the DMSO and performing an additional culture passage, which helps restore the cells’ viability and functionality [51].

The manner in which the MSCs are to be administered is strictly dependent on the phase of the heart disease. This is related to the fact that the pathophysiological aspects of the myocardial environment determine the cells’ ability to home and to be retained [72].

In the acute phase, e.g., in fresh MI, the damaged tissue releases groups of signaling molecules that play a key role in cell-to-cell communication. These include chemokines, cytokines, and growth factors. They constitute a migratory signal for stem cells [73,74]. In such a clinical case, the preferred methods of MSC administration are often intracoronary (IC) infusion or intravenous infusion (IV). In the chronic phase of the disease, e.g., ischemic cardiomyopathy (ICM) or HF, chemotactic signals are absent or scarce, which means that direct administration of MSCs into the myocardium is required. In this case, administration in the form of transendocardial injection (TESI) or transepicardial injection is recommended [72,75].

## 4. Routes of Administration

Several methods of administering MSCs in cardiac patients have been developed. These methods differ from one another primarily in their degree of invasiveness, the rate of retention in the myocardium, and the precision of cell application. The manner in which MSCs are administered frequently affects therapeutic efficacy [76,77]. The main routes of MSC administration include IV, IC, TESI, and transepicardial delivery.

IV delivery in the form of an infusion is the least invasive and simplest method, owing to the administration technique used and the possibility of repeated MSC application. This method requires neither a surgical procedure nor cardiac catheterization. In this case, the MSCs are administered into the bloodstream via a venous catheter and are then transported together with the blood to the ischemic region thanks to chemotactic signals, which is described by the phenomenon of homing [78,79]. In the context of intravenous infusion of MSCs, this phenomenon denotes a multistage process by which the MSCs independently find their way to the area of the heart affected by injury or inflammation [80]. Despite MSCs possessing a natural ability to migrate to the heart, this process is inefficient in the case of intravenous delivery [81]. This is related to the fact that a significant proportion of the administered cells become trapped in the capillaries of the lungs, liver, or spleen. This reduces the number of MSCs reaching the heart, which constitutes a significant disadvantage of this method. This phenomenon is referred to as the pulmonary first-pass effect [82,83]. Despite the low retention of MSCs in the heart, this method probably improves left ventricular function through a systemic anti-inflammatory action [84].

Another method used is IC, which consists of administering MSCs directly into the coronary arteries that supply the damaged area of the myocardium. The main advantages of this method include, above all, the minimal invasiveness of the procedure and the direct application of a large dose of MSCs to the damaged area of the heart. The MSCs are administered by means of a special balloon catheter introduced near the area of ischemia [76,85]. With this method, either a technique that maintains blood flow in the coronary vessels or a technique with flow blockade, the so-called “stop-flow,” is used. This technique consists of temporarily inflating the catheter balloon inside the vessel, which blocks blood flow and thereby facilitates the adhesion and migration of MSCs into the tissue, so that the rapid washout of cells is minimized [75,86]. Unfortunately, IC delivery is associated with a risk of microvascular embolization and microinfarction, which may result from the infusion of an excessive number of MSCs as well as from the administration of cells with an excessively large diameter [87,88,89]. It is also worth mentioning that in the case of high arterial pressure, washout of MSCs may occur, thereby limiting their retention [90]. The meta-analyses conducted indicate that IC infusion statistically significantly improves the left ventricular ejection fraction (LVEF), and also significantly affects myocardial viability after acute MI when this method of MSC administration is applied [17,91].

TESI involves the direct delivery of MSCs into the myocardium. This method involves minimally invasive MSC delivery using a catheter equipped with a needle, which is introduced via a peripheral artery into the cardiac cavity [92,93]. This approach also uses the NOGA system, which builds 3D electroanatomical maps of the heart. These maps allow accurate assessment of myocardial viability and identify ischemic and scarred regions. Above all, they enable precise targeting of the MSC delivery site. Such an advanced system analyzes the electrical activity of the myocardium and the mechanical properties of the cardiac wall, and accurately locates the position of the catheter tip within the cardiac cavity. This makes it possible to deliver MSCs in a targeted manner directly to the damaged but viable areas of the myocardium that have the greatest regenerative potential. The use of the NOGA system also improves the retention of MSCs in cardiac tissue [76,92]. In the case of the TESI method, a series of several to a dozen or so microinjections of MSCs is most often performed directly into the wall of the left ventricle [94]. Clinical studies have shown that TESI may contribute to reducing the area of the post-infarction scar, improving the LVEF, locally improving myocardial contractility, and reducing cardiac remodeling [92,94,95]. It should be borne in mind, however, that the above method may be associated with the risk of arrhythmia and perforation of the cardiac wall [90,92].

Transepicardial injection, like TESI, consists of delivering MSCs into the myocardium, but by means of a direct puncture of the heart from the outside. It is a more invasive method, as it requires open-heart surgery, and this procedure is usually applied as a supplement during cardiac surgical operations, e.g., during bypass procedures or thoracoscopy [81,96]. It should be noted that transepicardial injection overcomes the limitations of the TESI method, which include MSC washout and a low homing rate [90]. In addition, this delivery method does not require specialized imaging equipment, as the ischemic area is directly accessible during the procedure. Before the operation, the areas into which the MSCs are to be applied are imaged using echocardiography or magnetic resonance imaging (MRI) [81]. Subsequently, the MSCs are administered directly into the myocardium from the epicardial side, into the scar tissue or its border zone [96]. Despite the reliability of this method and the ease of surgical exposure, transepicardial injection requires an invasive procedure, which means that this type of MSC application is not frequently chosen by clinicians [81]. This method is also associated with an uneven distribution of MSCs within the infarct area, which may affect myocardial function. Intramyocardial injections may damage cardiac tissue and carry a risk of ventricular perforation. It is also worth mentioning the problem associated with leakage of MSCs from the injection site due to the cyclical contractions of the heart, which likewise constitutes a significant disadvantage of this method [90].

## 5. Strategies for Enhancing Cell Potential and Their Retention

**Figure 2:**
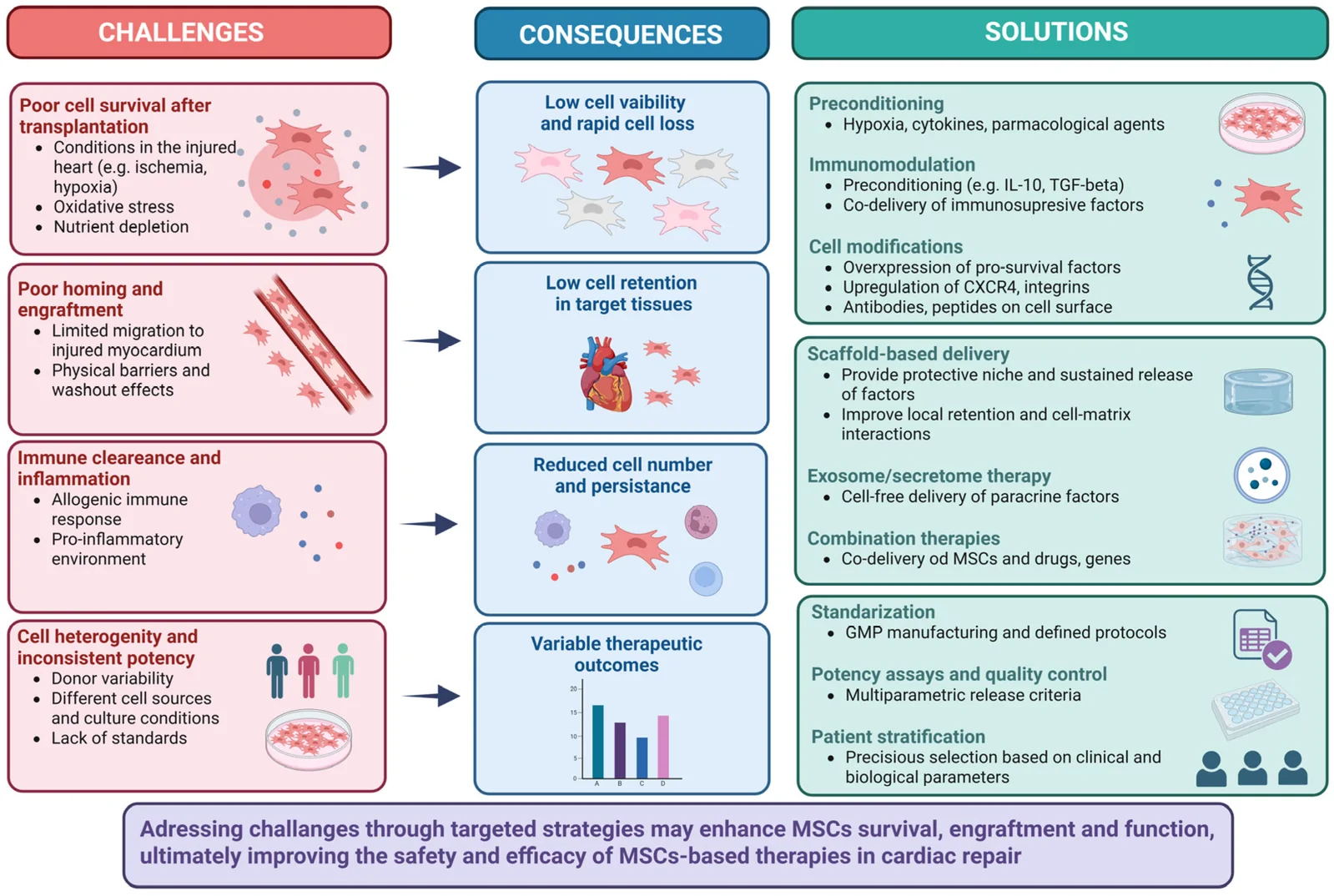
Challenges, consequences, and emerging solutions to improve MSC-based therapy in cardiac repair. Following administration, MSCs face multiple challenges, including ischemic and inflammatory conditions, oxidative stress, limited homing to the injured myocardium, immune recognition, and donor-to-donor variability. These obstacles reduce cell survival, retention, and long-term persistence, ultimately leading to inconsistent therapeutic efficacy. Novel strategies aimed at enhancing MSC performance include preconditioning approaches, immunomodulatory interventions, genetic engineering, biomaterial-assisted delivery, cell-free secretome therapies, combinatorial treatments, and the implementation of standardized manufacturing, potency testing, and patient-selection criteria. Created in BioRender. Deptuła, M. (2026) https://BioRender.com/2nvieo3.

Prior to transplantation, MSCs may be subjected to preconditioning (priming), which consists of enhancing the cells’ potential by means of advanced ex vivo manipulation techniques (Figure 2). The aim of MSC preconditioning is to minimize the negative impact of the ischemic environment of the heart, which includes, among others, oxidative stress, in order to increase retention, cell survival, and their reparative activity [90,97]. Strategies for enhancing the therapeutic potential of MSCs and increasing their retention in the myocardium are one of the key areas of research in regenerative cardiology.

Hypoxic preconditioning is one of the best-studied methods of strengthening MSCs before their use in a given clinical case. This method mimics natural physiological tissue niches by culturing MSCs under conditions of reduced oxygen concentration (usually 1–5% O_2_). The mechanism of MSC preconditioning consists of the activation of signaling pathways dependent on the hypoxia-inducible factor 1-alpha (HIF-1α) and Akt kinase, which increases resistance to apoptosis induced by hypoxia and lack of adhesion [46,98]. As a result, overexpression of chemokine receptors occurs, and thus the ability of MSCs to home to damaged tissue is improved, while the secretion of angiogenic factors also increases [99]. It has been shown that IC injection of autologous bone marrow-derived mononuclear cells subjected to hypoxic preconditioning significantly delays the process of left ventricular remodeling after infarction [100].

In the case of pharmacological and chemical preconditioning, the MSCs are pre-incubated with drugs or chemical molecules intended to increase their metabolic resistance [90]. The most commonly used are drugs that protect against oxidative stress, e.g., trimetazidine (which increases the expression of Bcl-2 and Akt), diazoxide (which activates ATP-dependent potassium channels), and antioxidants (vitamin E). Mesenchymal stem cells preconditioned with trimetazidine promote neovascularization of hearts under hypoxia/reoxygenation injury [101,102,103]. Statins are also used and are thought to enhance paracrine activity through the release of exosomes rich in lncRNA H19, which stimulates angiogenesis. In the case of incubation with statins, e.g., atorvastatin, an improvement in the survival, migration, and differentiation capacity of MSCs occurs [104,105].

It is also worth mentioning chemical compounds that mimic the action of hypoxia, even though the cells are cultured at a normal oxygen concentration. Such compounds, called hypoxia mimetics, elicit a response characteristic of hypoxia without the need to place the cells in a hypoxic chamber. They induce the accumulation of HIF-1α under normoxic conditions, producing effects similar to physical hypoxia. Hypoxia mimetics inhibit the action of prolyl hydroxylases, so that HIF-1α remains active even at a normal oxygen concentration [106,107]. The most commonly used chemical compounds in MSC therapy are cobalt chloride (CoCl_2_), deferoxamine (DFO), or dimethyloxalylglycine (DMOG) [108,109,110]. Hypoxia mimetics increase the survival of MSCs, improve their retention, and also enhance their anti-apoptotic effect [111].

Cytokine and growth factor stimulation is also used to enhance the potential of MSCs. This is one of the methods aimed at increasing the therapeutic potential of MSCs before their transplantation. This type of preconditioning consists of the brief exposure of the cells to specific signaling molecules that activate pathways responsible for survival, migration, angiogenesis, and immunomodulation [112]. The most commonly used cytokines include interferon-gamma (IFN-γ), tumor necrosis factor-alpha (TNF-α), and interleukin-1 beta (IL-1β) [113]. As for growth factors, the most commonly used combinations are VEGF, IGF-1, HGF, and basic fibroblast growth factor (FGF-2) [98,107].

Contemporary strategies for increasing the potential of MSCs in cardiology also focus on the transition from standard monolayer (2D) culture to three-dimensional (3D) structures, such as spheroids [2]. Culturing MSCs in the form of 3D spheroids increases their resistance to hypoxia through the activation of hypoxia pathways and the enhanced secretion of VEGF and stromal cell-derived factor-1 (SDF-1) [114,115]. MSCs in such cultures exhibit a stronger paracrine action, greater resistance to oxidative stress, and consequently higher therapeutic efficacy [116]. It has also been shown that co-culturing MSCs with cardiac cells in spheroids may additionally improve myocardial function and reduce the infarct area [117].

It is also worth mentioning that an important issue in increasing the survival of MSCs and enhancing their retention at the target site is the use of various biomaterial carriers. Biomaterials and scaffolds in MSC-based therapy are one of the key strategies for increasing regenerative efficacy in the myocardium. In this method, MSCs are seeded onto a 3D carrier structure that mimics the extracellular matrix [90,118]. In MSC-based therapy, biomaterials can be used without forming a scaffold, or as ready-made 3D scaffolds onto which MSCs are seeded prior to implantation [119]. Scaffolds constitute a key element of tissue engineering, serving as physical support and a protective microenvironment for the transplanted MSCs [120]. Modern scaffolds can be enriched with systems for the controlled release of drugs, genes, or growth factors, which allows for local modification of the microenvironment toward a pro-regenerative one [121]. Biomaterials and scaffolds provide the cells with the necessary adhesive signals, which significantly increases their survival in the hostile post-infarction environment.

In cardiac tissue engineering, three main groups of biomaterials are used: natural polymers, synthetic polymers, and decellularized scaffolds. Natural polymers used include, among others, collagen, fibrin, alginate, chitosan, hyaluronic acid, and Matrigel-type gels. They exhibit high biocompatibility and biodegradability [122,123]. As for synthetic polymers, poly(lactic-co-glycolic acid) (PLGA), polycaprolactone (PCL), and poly(glycerol sebacate) (PGS) are used, which provide greater control over the mechanical properties of the material and the rate of its degradation [124,125]. Decellularized scaffolds are created by removing cells from natural tissues (e.g., porcine myocardium or human pericardium), which makes it possible to preserve the 3D structure of the extracellular matrix and its mechanical properties [126].

An important issue in cardiac tissue engineering using MSC cell therapy is the manner of cell delivery with the use of biomaterials. The simplest and most commonly used form is injectable hydrogels. MSCs are mixed with a hydrogel and subsequently injected directly into the myocardium. After injection, the hydrogel hardens within the tissue, forming a mechanical barrier that prevents the washout of cells [90,127]. Another, more advanced method is the use of cardiac patches. These are thin biological or biomaterial structures with embedded MSCs, applied directly onto the area of the scar. They provide physical support for the weakened muscle and can be enriched with growth factors [128]. Microencapsulation of MSCs in microspheres also constitutes an important method of cell delivery. The MSCs are enclosed in semipermeable microcapsules made of a biomaterial (e.g., alginate), which creates a protective microenvironment around the cells. Microencapsulation of MSCs protects them from attack by the host immune system, while at the same time allowing the free diffusion of therapeutic paracrine factors [129,130].

To overcome the hostile microenvironment of the ischemic myocardium and maximize the paracrine impact of cMSCs, bioengineering approaches combining cMSCs with functionalized biomaterials and targeted delivery vehicles have been developed [32,131]. Encapsulation of cMSCs within biocompatible porous scaffolds provides protective physical niches that prevent anoikis while enabling sustained, localized release of therapeutic paracrine mediators and extracellular vesicles (EVs) [132]. EVs secreted by human cardiac stromal cells contain distinct microRNA and protein cargos that directly upregulate protective anti-apoptotic and autophagic signaling pathways in native cardiomyocytes under hypoxic stress, preserving cell viability and reducing infarct size [34,133]. Furthermore, combining biomaterial encapsulation with nanocarrier-based targeted delivery systems allows for site-specific accumulation of MSC secretomes or therapeutic EVs within the ischemic border zone, bridging cell-free paracrine biology with advanced tissue engineering to achieve superior cardiac repair [131].

Molecular modifications of MSCs in cardiac therapy consist of their deliberate modification at the level of DNA, mRNA, or protein expression, in order to increase therapeutic efficacy following the administration of MSCs to the damaged myocardium. The main goal of MSC genetic engineering is to strengthen their natural properties: survival, migration, angiogenesis, and anti-inflammatory action [134]. The most common target of molecular modifications is the overexpression of the Akt1, Bcl-2, or HO-1 genes, which drastically reduces MSC apoptosis after administration [134]. Improving homing and accelerating the migration of MSCs to the infarct area is also an important issue that is a target of molecular modifications, through the introduction of genes for chemokine receptors (e.g., CXCR4, CCR1) [134,135]. Increasing the clinical efficacy of MSCs also involves modifications of genes for VEGF or HGF, which increases the angiogenic and antifibrotic potential of the cells [90,136].

## 6. Imaging and Monitoring of the Transplant

Imaging and monitoring of the MSC transplantation process are important technical aspects that allow for the precise delivery of the therapeutic agent to the damaged myocardium and the assessment of its fate and efficacy after the procedure. Monitoring of MSCs is necessary because, after their administration, a significant number of cells do not reach the heart, some undergo apoptosis, and only a small percentage of them remain in the target tissue. In view of the above, it is important to monitor them in order to estimate their therapeutic efficacy [137,138].

Precise localization of the injection site is essential, especially in the case of transendocardial administration of MSCs, in order to distinguish viable but dysfunctional tissue from inactive fibrous scar [93]. Intraprocedural navigation makes it possible to precisely deliver the cells to the most therapeutically active areas of the myocardium, which increases their retention and efficacy. As already mentioned, 3D electromechanical mapping, called the NOGA^®^ system, is the most advanced navigation technology used in transendocardial injections. This system creates a 3D map of the left ventricle by integrating electrical and mechanical signals, enabling precise identification of the peri-infarct region, which has the highest regenerative potential [76,139]. In transepicardial administration, navigation is based on the direct visualization of the scar by the surgeon during the operation [81]. In IC methods, navigation is simpler and is based on standard 2D X-ray fluoroscopy in order to position the catheter in the infarct-related artery [140].

Cell tracking after transplantation is crucial for assessing MSC biodistribution, migration, retention, and survival. These parameters represent major limitations of cardiac cell therapy, as only a small fraction of administered cells is retained within the target myocardium, and long-term cell survival is generally very low, often approaching 1% or less [50,141]. One commonly used method is the dual labeling of cells with superparamagnetic iron oxide (SPIO) nanoparticles, which allows for their visualization in magnetic resonance imaging and the precise determination of their anatomical location in the myocardium [137]. MSCs can also be labeled with radioisotopes, which allows for the assessment of their redistribution in the body by means of scintigraphy or positron emission tomography (PET) [142]. In preclinical studies, reporter genes (e.g., luciferase) are also used, which allow for the non-invasive monitoring of the viability and proliferation of cells in vivo over a longer period of time. Such labeling produces the phenomenon of bioluminescence, which is exploited in animal models [137].

Assessment of transplant efficacy requires the use of advanced medical imaging tools in order to monitor structural and functional changes in the heart. Cardiac magnetic resonance imaging (CMR) is considered the gold standard in the assessment of chamber volumes and the ejection fraction, owing to its high precision. This method also allows for the most accurate assessment of the reduction in scar size after cell therapy [39]. Echocardiography is one of the most widely used imaging modalities for monitoring cardiac structure and function because of its broad availability, non-invasive nature, and relatively low cost. However, conventional echocardiographic assessment remains operator-dependent and may be affected by intra- and interobserver variability. Stress echocardiography can additionally provide information on myocardial ischemia, viability, and functional reserve [143]. Monitoring also includes regular measurements of the concentration of the N-terminal pro–B-type natriuretic peptide (NT-proBNP) biomarker, which is an indicator of ventricular overload and the progression of HF [144]. Proper selection of navigation and monitoring methods allows not only for increasing the safety of the procedure, but also for gathering reliable data necessary for the standardization of clinical protocols.

## 7. Clinical Applications of MSCs in Heart Diseases

**Figure 3:**
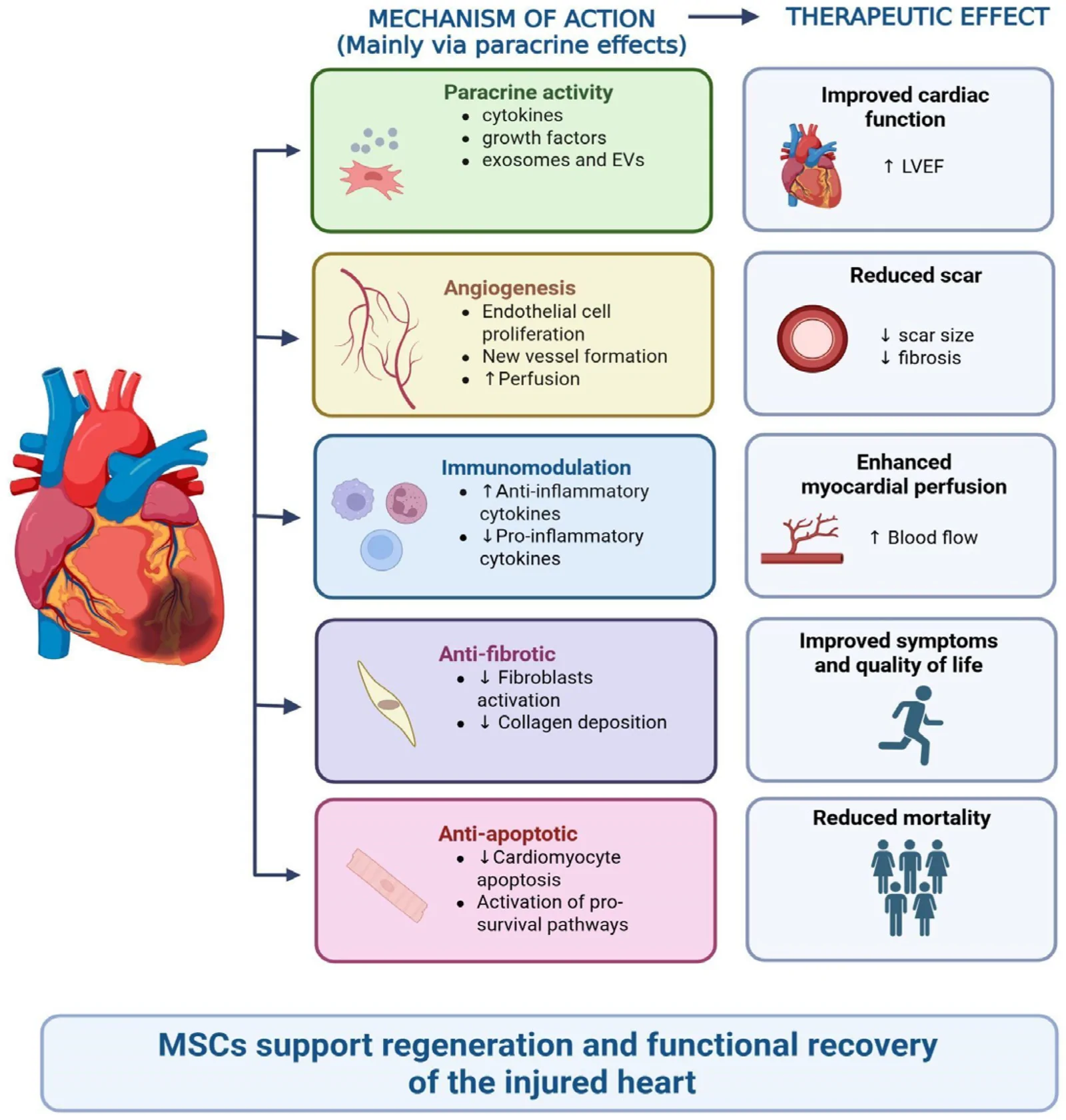
Mechanisms of action and therapeutic effects of MSCs in cardiac regeneration. Following administration, MSCs support tissue regeneration primarily via paracrine mechanisms rather than direct differentiation into cardiomyocytes. Secreted bioactive molecules, including cytokines, growth factors, and extracellular vesicles, stimulate neovascularization, enhance tissue perfusion, regulate immune and inflammatory responses, suppress fibroblast activation and extracellular matrix deposition, and activate pro-survival pathways in resident cardiac cells. These combined actions facilitate structural and functional recovery of the injured myocardium, resulting in improved left ventricular function, reduced fibrotic remodeling, enhanced myocardial perfusion, and better clinical outcomes. ↑ means an increase, ↓ means a decrease. Created in BioRender. Deptuła, M. (2026) https://BioRender.com/o3lv1um.

MSC-based therapies are therefore being investigated for myocardial repair and functional recovery (Figure 3). The following sections summarize the main cardiovascular indications for MSC therapy according to the available evidence. Consistent with ICD-11 [145], diseases of the coronary arteries are discussed separately from IHD.

### 7.1. Ischemic Heart Diseases

Ischemic heart disease spans stable angina to acute coronary syndromes (unstable angina and acute MI and is the leading cause of cardiovascular death [5,17]. IHD is characterized by an imbalance between myocardial oxygen supply and demand, leading to MI, which may impair cardiac function [146,147]. The vascular mechanisms underlying myocardial ischemia, as well as acute MI-the most severe clinical manifestation of IHD-are discussed in detail in this section.

Sustained or repeated ischemia induces oxidative stress, mitochondrial dysfunction, inflammation, and cardiomyocyte death. Together, these processes promote adverse remodeling and progressive contractile dysfunction [148]. Because the regenerative capacity of the adult myocardium is limited, the cumulative loss of viable cardiomyocytes contributes to long-term deterioration of cardiac performance and increases the risk of HF [4,149]. This argues for regenerative strategies that restore not only perfusion but also tissue integrity and function.

Percutaneous coronary intervention (PCI) and coronary artery bypass grafting (CABG) restore epicardial blood flow and relieve ischemia. However, neither approach replaces lost cardiomyocytes or reverses established myocardial damage, and residual microvascular dysfunction often persists [7,17]. Many patients therefore remain symptomatic and continue to deteriorate despite successful revascularization [147].

A clinically important and therapeutically challenging subgroup within IHD comprises so-called “no-option” patients—individuals with refractory angina who remain symptomatic despite optimal medical therapy yet are ineligible for further revascularization because of diffuse multivessel disease or previous failed interventions [150]. In this population, cell-based therapies have primarily been investigated as a strategy to alleviate ischemic symptoms and improve functional status. A meta-analysis of randomized controlled trials demonstrated significant reductions in both all-cause mortality (odds ratio 0.24; 95% confidence interval (CI) 0.10–0.60) and major adverse cardiac events (odds ratio 0.41; 95% CI 0.25–0.70), together with improvements in anginal frequency, Canadian Cardiovascular Society angina class, and exercise tolerance [150].

Acute MI is the most severe, time-critical form of IHD. It usually follows plaque rupture or erosion, which activates platelets, forms a coronary thrombus, and abruptly cuts off myocardial blood supply [7,17]. The resulting ischemia triggers cardiomyocyte necrosis. The extent of myocardial injury depends on the duration of coronary occlusion, the affected vascular territory, and collateral circulation. A large transmural infarct can destroy on the order of a billion cardiomyocytes, which the adult heart cannot replace, leaving an inert fibrotic scar [151].

Reperfusion is itself a source of injury. Although prompt restoration of flow limits infarct size and is the cornerstone of acute MI care, it also triggers an inflammatory response—ischemia/reperfusion (I/R) injury—that extends damage beyond the originally ischemic zone [152]. Post-infarction inflammation is driven by innate immune activation involving the NLRP3 inflammasome, inflammatory cell infiltration, and cytokine release. These processes contribute to infarct expansion, fibrotic scar formation, and adverse ventricular remodeling [152]. Because broadly suppressing post-MI inflammation can impair healing, the timing of any intervention matters. Following infarction, the myocardium undergoes a temporally organized remodeling process characterized by cardiomyocyte hypertrophy, interstitial fibrosis, and compensatory changes in non-infarcted segments. The balance among these adaptive and maladaptive responses ultimately determines whether the heart recovers function or progresses to HF [153,154].

After MI, MSCs may reduce cardiomyocyte death in the peri-infarct zone, dampen acute inflammation, promote neovascularization, and limit adverse left-ventricular remodeling [149,155]. Principal clinical endpoints evaluated in this context include changes in LVEF, infarct size on cardiac magnetic resonance imaging, and rates of major adverse cardiovascular events (MACE) [156].

Although MI and chronic IHD differ in their pathophysiology, MSC-based therapies target several mechanisms common to both conditions. Cardiac-derived mesenchymal stem cell-like cells (CMSCLCs) differ from BM-MSCs, including their limited osteogenic and chondrogenic differentiation [32]. Their extracellular vesicles protected human cardiomyocytes derived from induced pluripotent stem cells against hypoxia/reoxygenation injury more effectively than BM-MSC-derived vesicles in vitro [133]. Encapsulation of cardiac mesenchymal cells in a porous scaffold prolonged cell survival and retention and preserved ejection fraction in a mouse model of MI [132]. These findings remain preclinical.

MSCs are widely used in cardiac regenerative therapies, primarily in intramyocardial (IM) and transmyocardial injections. In contrast, intravenous therapies are less commonly used and have been less extensively studied in in vitro and in vivo models [157]. Levit et al. [158] used human MSCs encapsulated in sodium alginate and then suspended in a hydrogel. They administered these constructs to rats in which MI was induced by ligation of the left anterior descending artery. It was observed that the ejection fraction after MI, as measured by CMR, improved significantly in animals treated with encapsulated MSCs. Additionally, scar size was significantly reduced after 28 days, and microvascular density increased in the peri-infarct area. However, the constructs had no effect on the number of proliferating cardiomyocytes or apoptotic cells. Li et al. [159] compared two methods of MSC administration in a hydrogel composed of an extracellular matrix in mice that had suffered a heart attack. IM injections were performed into the myocardium, while intrapericardial (IPC) administration was conducted into the pericardial cavity. Short-term (2 weeks) and long-term (6 weeks) echocardiography confirmed an improvement in cardiac function of approximately 40% following IPC administration compared to the IM method (4%). Additionally, improved cell proliferation and vascular regeneration, as well as reduced apoptosis in the myocardium, were observed following intracardiac administration. MSCs have also been used in clinical trials. Hare et al. [160] administered human MSCs (hMSCs) to 39 patients with MI following reperfusion. Twenty-one patients received a placebo. A reduction in the number of episodes of ventricular tachycardia and an improvement in forced expiratory volume were observed in patients treated with hMSCs. Additionally, the administration of the cells increased the LVEF. In contrast, Gao et al. [161] administered WJ-MSCs or a placebo to the infarcted artery in 116 patients with acute ST-segment elevation myocardial infarction following successful reperfusion therapy. An increase in myocardial viability PET, global LVEF, and perfusion in the infarct area was observed following the administration of WJ-MSCs.

### 7.2. Coronary Artery and Coronary Microvascular Diseases

Coronary artery disease (CAD) results from atherosclerotic plaque in the epicardial arteries, which narrows the lumen and impairs perfusion, producing a clinical spectrum from stable angina to acute coronary syndromes and ICM [7,17]. Atherosclerosis is fundamentally an inflammatory disease of the arterial wall: endothelial dysfunction and subintimal lipid accumulation are followed by monocyte recruitment, foam-cell formation, and fibro-inflammatory plaque growth [162,163].

Myocardial ischemia is not caused by epicardial obstruction alone. Up to half of patients who undergo invasive angiography for angina, or have non-invasive evidence of ischemia, are found to have non-obstructive coronary arteries. This has established coronary microvascular dysfunction (CMD) as a major, independent cause of MI [162,164]. CMD encompasses a spectrum of structural and functional alterations in the coronary microcirculation, affecting vessels with a diameter below 500 μm. These changes include arteriolar remodeling, impaired endothelium-dependent vasodilation, enhanced vasoconstrictor responsiveness, and perivascular fibrosis, and they share key pathophysiological mechanisms with atherosclerosis, including chronic low-grade inflammation and endothelial dysfunction [164,165]. These abnormalities limit the ability of the coronary circulation to augment blood flow when myocardial oxygen demand increases. As a result, patients may develop ischemia with non-obstructive coronary arteries (INOCA), angina with non-obstructive coronary arteries (ANOCA), or MI with non-obstructive coronary arteries (MINOCA). INOCA affects an estimated 3–4 million individuals and is associated with an elevated cardiovascular risk [162,166].

CMD is not limited to non-obstructive coronary disease; it also contributes to obstructive CAD (where it persists after successful epicardial revascularization), non-ischemic cardiomyopathies, takotsubo syndrome, and HF with preserved ejection fraction [162]. Although CMD is now recognized as clinically important, drug options for it remain few and are poorly supported by randomized outcome trials [167].

MSCs are a plausible candidate in CMD: they promote endothelial repair through paracrine VEGF, FGF-2, and HGF, attenuate vascular inflammation, reduce perivascular fibrosis, and stimulate neovascularization [17]. CD34+ endothelial progenitor cells have also been evaluated in pilot studies involving patients with INOCA and endothelial-independent CMD. Unlike MSCs, these cells promote vascular repair primarily through direct endothelial regeneration rather than paracrine signaling. In the ESCaPE-CMD trial, CD34+ cell therapy significantly improved coronary flow reserve, angina frequency, and quality of life [166]. Together, these approaches suggest that cell therapy could be matched to the dominant disease mechanism [17,168].

Several recent studies have examined MSCs and their secretome-derived products in coronary artery and microvascular disease. Mostafavi et al. [169] described MSC-derived exosomal microRNAs (exo-miRs) in preclinical models of CAD. Of 56 preclinical CAD models, 17 were concerned with ischemia/reperfusion, 4 with ischemia, and 1 with hypoxia. In vitro studies focused on the anti-inflammatory and anti-apoptotic effects of exosomes under hypoxic conditions. In contrast, animal studies focused on improving cardiac function, reducing left ventricular end-diastolic and end-systolic diameters, and decreasing the extent of fibrosis. It has been demonstrated that exosomal miRNAs derived from various MSC sources have a protective role in regulating impaired signaling in CAD. Wen et al. [170] investigated the effect of miRNA-144 derived from exosomes produced by BM-MSCs on rat cardiomyocytes under hypoxic conditions. A protective effect of miRNA-144 against cardiomyocyte apoptosis was observed. Additionally, it was found that the pro-apoptotic gene *PTEN* (phosphatase and tensin homolog deleted on chromosome 10) is a target of miRNA-144. Clinical trials using MSCs have also been conducted. Heldman et al. [171] performed transendocardial injections of autologous MSCs and whole bone marrow mononuclear cells (BMCs) in patients with ICM. MSCs or BMCs were administered to 19 patients, while 21 patients received a placebo. No patient experienced any serious adverse events after 30 days of follow-up. The 6-min walk distance increased, and the infarct size as a percentage of left ventricular mass decreased following MSC administration. The left ventricular volume and ejection fraction remained unchanged.

### 7.3. Heart Failure

Heart failure is the common endpoint of many cardiovascular disorders and now affects more than 64 million people worldwide [4]. HF is a clinical syndrome characterized by structural or functional cardiac abnormalities that impair ventricular filling or ejection. These changes reduce the heart’s ability to meet the metabolic demands of the body and manifest as symptoms such as dyspnea, fatigue, and fluid retention [4]. Despite guideline-directed medical therapy (renin–angiotensin–aldosterone inhibitors, beta-blockers, mineralocorticoid receptor antagonists, and SGLT2 inhibitors) and device therapy (CRT and implantable cardioverter-defibrillators), mortality and hospitalization remain high [4]. Current treatments reduce disease progression but do not reverse the loss of functional myocardium caused by fibrosis and cardiomyocyte loss.

HF progresses through the interaction of structural, neurohormonal, and immune mechanisms. Following myocardial injury, surviving cardiomyocytes undergo hypertrophy, while interstitial fibrosis and ventricular dilation gradually impair systolic and diastolic function. Persistent activation of the renin–angiotensin–aldosterone and sympathetic nervous systems further accelerates pathological remodeling [172,173]. Chronic, low-grade inflammation—sustained by interleukin-6, interleukin-1β, and TNF-α signaling—links the initial myocardial injury to progressive dysfunction. This can advance even when the LVEF is preserved [174]. These pathways represent potential therapeutic targets for MSC-based therapy.

Clinical interest has focused mainly on heart failure with reduced ejection fraction (HFrEF), especially in patients who stay symptomatic despite optimal medical therapy. This group carries the greatest burden of structural myocardial damage [17,149]. Randomized controlled trials have evaluated endpoints including 6-min walk test (6MWT) distance, Minnesota Living with Heart Failure Questionnaire (MLHFQ) scores, New York Heart Association (NYHA) functional class, and HF-related hospitalization rates [149,175,176]. Clinical evidence, however, remains mixed. In DREAM-HF, the largest randomized trial to date, a single transendocardial administration of allogeneic mesenchymal precursor cells was evaluated in 565 patients with high-risk HFrEF. Although the primary endpoint of recurrent decompensated HF events was not met, prespecified analyses demonstrated reductions in MI, stroke, and MACE, particularly among patients with elevated baseline inflammation [177]. These findings support inflammation as a therapeutic target, but efficacy on standard HF endpoints remains unproven.

MSCs were also tested for their activity in HF. Makkaoui et al. [178] created a rat model of pediatric right ventricular (RV) heart failure due to pressure overload by pulmonary artery banding (PAB) and delivered BM-MSCs to the RV free wall. Next, they performed proteome profiling and identified proteins significantly changed after PAB. Then analyzed the response to cell therapy and found that 72% of the proteome altered by PAB was fully or partially reversed by MSC treatment. The analysis showed that cell therapy may cause a decrease in cardiac adverse effects and reverse the proteome to a pre-disease state. Another study showed the use of UC-MSC sheets in rat models of IHD. The results showed that MSC sheets improved cardiac function and attenuated ventricular remodeling. The results were obtained through anti-fibrotic and pro-angiogenic mechanisms and inhibition of the PI3K/Akt/mTOR signaling pathway. What is more, the cell sheet promoted UC-MSC retention in the heart, which indicates that it is a promising strategy for clinical use [179]. MSCs were also applied in clinical trials in humans. The PRIME-HFrEF Trial was a single-center, randomized, placebo-controlled, investigator-initiated study (Clinical Trial Registry: ClinicalTrials.gov; registration number: NCT04992832; registration date: 5 August 2021) that evaluated the safety of three intravenous infusions of UC-MSCs administered at six-week intervals in patients with HF and reduced ejection fraction (HFrEF). The study enrolled 40 patients and additionally collected efficacy data. There was no significant difference in the incidence of serious adverse events between the control and study groups. UC-MSC administration did not significantly change ΔLVEF. However, a greater improvement in end-systolic volume (ΔRV end-systolic volume (RVESV)) was observed in the study group. Elevated D-dimer levels were also observed. This study demonstrates that UC-MSC use is safe for patients and improves RV volume; however, a transient increase in coagulation may potentially impair left ventricular systolic function [180]. A meta-analysis of 17 randomized controlled trials (1684 patients: 927 patients in the intervention and 757 in the control) examined the efficacy of MSCs in MACE and cardiac function indices in patients with chronic HF. The results suggest that MSCs may reduce mortality and improve clinical outcomes in patients with ischemic or dilated HF. They also improve left ventricular function (LVEF increase, left ventricular end-systolic volume (LVESV) and left ventricular end-diastolic volume (LVEDV) decrease), quality of life, and scarring [175]. Another analysis involving 12 clinical studies showed significant improvement in patients’ quality of life. There were no significant changes in LVEF, Global Circumferential strain, and the 6-Minute Walk Test. There was no increase in major adverse cardiac events [181]. Available data suggest that MSC source is important for the clinical outcome. A meta-analysis of 13 randomized controlled clinical trials (693 patients) showed that application of UC-MSCs improved LVEF, but no significant differences were observed for BM-MSCs. None of the cells improved 6-min walking distance. Nor did they cause any changes in the occurrence of major adverse cardiac events. However, rehospitalization rates were lower with BM-MSCs [182].

### 7.4. Cardiomyopathies

Cardiomyopathies are myocardial disorders with structural and functional abnormalities not attributable to CAD, hypertension, valvular disease, or congenital heart disease. The 2023 European Society of Cardiology (ESC) Guidelines refine this definition using a phenotype-based classification [183]. According to these guidelines, cardiomyopathies are classified into hypertrophic, dilated, restrictive, arrhythmogenic, and non-dilated left ventricular phenotypes, each a distinct morphological and functional entity [183]. Ischemic cardiomyopathy is not classified as a primary cardiomyopathy in the current ESC framework. Even so, it is the leading cause of chronic ventricular dysfunction and systolic HF worldwide, and it has been the most studied indication for MSC-based therapy, accounting for most completed randomized trials in the field [17,149,172]. For this reason, the following sections focus on ICM and non-ischemic dilated cardiomyopathy (NIDCM), the two main clinical targets of MSC-based therapy.

In ICM, tissue loss after infarction combines with reduced coronary reserve and hibernating but potentially viable myocardium—a setting suited to regenerative approaches that limit fibrosis, promote new vessels, and preserve remaining myocardium [172].

NIDCM is an important target of MSC-based investigation, with early clinical studies suggesting potential benefit [17,176]. NIDCM is characterized by left ventricular dilatation and systolic dysfunction, frequently accompanied by RV involvement and myocardial fibrosis, in the absence of CAD sufficient to explain the phenotype [183].

An early systematic evaluation of cell therapy in NIDCM was provided by a meta-analysis conducted by Marquis-Gravel et al. in 2014 [184], which pooled data from four randomized trials involving 244 patients. The analysis demonstrated a significant improvement in LVEF compared with control groups, providing early proof-of-concept for cell-based therapies in this population, although most studies employed bone marrow mononuclear cells rather than purified MSCs [184].

Subsequent studies evaluating purified MSC populations expanded these observations. The POSEIDON-DCM trial [176] evaluated transendocardial administration of bone marrow-derived MSCs in 37 patients with NIDCM, comparing allogeneic and autologous preparations. Significant improvements from baseline in LVEF, six-minute walk distance, and quality-of-life scores were observed in the allogeneic group, whereas the corresponding changes in the autologous group were not statistically significant. The between-group difference in LVEF improvement was not statistically significant [176]. This was the first randomized study to evaluate purified MSC therapy in patients with NIDCM. The results demonstrated its feasibility and suggested potential clinical benefit [176]. These results justify further study of MSC therapy in non-ischemic cardiomyopathy.

More recent studies have provided additional evidence regarding the efficacy and safety of MSC therapy in ischemic and non-ischemic cardiomyopathy and on new delivery methods.

In 2024, a review of clinical trials evaluating the safety and efficacy of MSCs in the treatment of ICM was published. An increase in LVEF of 5.75% was observed, compared to 3.19% in the control group. In addition, the 6-min walk test and tests designed to assess exercise tolerance and quality of life showed improved results for patients in the treatment group. Serious adverse events following MSC administration were reported in 33 studies. A total of 80 deaths were observed, of which 41.3% were deemed unrelated to the intervention itself. The cause of death was not reported for 40% of the deaths [185]. The study, numbered NCT02467387, evaluated the use of MSCs in patients with non-ischemic cardiomyopathy who had an LVEF below 40% and no hyperintensity on cardiac magnetic resonance imaging. After MSC treatment, the LVEF and ventricular volumes were similar to baseline values. However, the use of MSCs increased the 6-min walk distance and improved functional status and clinical parameters of cardiomyopathy [186]. In addition to standard MSC administration, stem cells can be supplied in the form of sheets that detach from the substrate at 32 °C without enzymatic digestion. BM-MSC sheets were transplanted into rats in an ICM model, resulting in a 6% improvement in LVEF 28 days after transplantation. Additionally, BM-MSC sheets were tested in a porcine ICM model using an immunosuppressive drug. After 8 weeks, an increase in LVEF of approximately 20% was observed [187].

### 7.5. Myocarditis

Myocarditis is defined as inflammatory injury of the myocardium, with a globally estimated incidence of approximately 4 to 14 cases per 100,000 persons per year [188]. Reported prevalence estimates range from 10.2 to 105.6 per 100,000 worldwide [189], but the disease burden is likely underestimated because of the heterogeneous and often subclinical clinical presentation [190].

Myocarditis has infectious, autoimmune, and drug-induced causes [191]. Viral infections are the leading cause of myocarditis in industrialized countries. The most common pathogens include Coxsackievirus B, influenza virus, parvovirus B19, and SARS-CoV-2. Rare cases of myocarditis have also been reported following mRNA COVID-19 vaccination [188]. Autoimmune diseases and immune checkpoint inhibitors are among the major non-infectious causes [188]. Myocarditis typically evolves through several pathogenic phases. The disease initially involves an innate immune response and direct viral cytopathic injury. This is followed by adaptive immune cell infiltration and cytokine-mediated myocardial damage. In some patients, inflammation persists, ultimately leading to chronic myocardial inflammation and inflammatory dilated cardiomyopathy [191]. Approximately 25% of patients develop left ventricular systolic dysfunction, ventricular arrhythmias, or acute HF, and acute myocarditis complicated by hemodynamic instability carries an approximately 28% rate of mortality or heart transplantation within 60 days [188].

MSCs are attractive candidates for myocarditis because they suppress T-cell activation, promote anti-inflammatory macrophage polarization, and reduce the release of pro-inflammatory cytokines [7]. Persistent inflammation and immune dysregulation also promote ventricular remodeling and progression to inflammatory dilated cardiomyopathy [190,191]. The immunomodulatory effects of MSCs provide a rationale for investigating whether this process can be attenuated [7]. Although clinical evidence remains limited, preclinical studies in autoimmune and viral myocarditis models have consistently demonstrated the anti-inflammatory effects of MSCs.

The biological activity of MSCs in myocarditis is mainly based on the secreted immunomodulatory proteins and growth factors. Many studies involve not only MSCs, but also their exosomes [192]. MSCs of various origins were tested for their therapeutic effect in myocarditis. BM-MSCs were proven to significantly attenuate myocardial injury in a rat model of acute myocarditis. In this study, these cells increased angiogenesis and reduced inflammation, partly through their paracrine effect [193]. Similarly, intravenous administration of allogenic fetal membrane-derived MSCs resulted in attenuated myocardial dysfunction and inflammation, which was mainly caused by the suppression of T lymphocyte activation [194]. Furthermore, Miteva et al. [195] showed that MSCs reduced myocardial inflammation by suppressing infiltration of pro-inflammatory cytokines and at the same time promoting infiltration of anti-inflammatory monocytes. What is more, MSCs may be used to deliver bioactive molecules for the treatment of myocarditis. For example, BM-MSCs overexpressing IL-10 were injected into mice with autoimmune myocarditis. The results showed enhanced immunosuppressive activity of these cells and improved therapeutic effects. The anti-troponin I level and expression of pro-inflammatory cytokines (IL-1beta, TNF-alpha, IL-6, IL-17) were reduced, while the infiltration of M2 macrophages in the heart increased [196].

### 7.6. Pulmonary Heart Disease or Diseases of Pulmonary Circulation

Pulmonary hypertension (PH) is a progressive cardiopulmonary syndrome hemodynamically defined by a mean pulmonary arterial pressure exceeding 20 mmHg at rest on right heart catheterization [197]. The current classification encompasses five clinically and pathophysiologically distinct groups, with pulmonary arterial hypertension (PAH; WHO Group 1) representing the most extensively studied and specifically targeted form [197,198,199]. In PAH, the small pulmonary arteries undergo progressive obliterative remodeling driven by endothelial dysfunction, smooth-muscle hyperproliferation, and inflammatory vasculopathy. This raises pulmonary vascular resistance and RV afterload, and survival remains poor despite several approved therapies [199].

The molecular pathogenesis of PAH reflects a complex interplay among chronic vascular inflammation, dysregulated angiogenesis, metabolic remodeling, genetic and epigenetic alterations (especially BMPR2 mutations), and sex hormone-mediated susceptibility [198]. Current therapies target the endothelin, nitric oxide, and prostacyclin pathways, while sotatercept acts as a ligand trap for activins and related growth factors, modulating a distinct pathway involved in vascular remodeling [200]. Despite these therapeutic advances, some patients have an inadequate response to medical therapy, supporting continued investigation of additional therapeutic approaches [200].

MSC therapy has emerged as a promising therapeutic approach for PAH. Experimental studies show that MSCs suppress pulmonary arterial smooth-muscle proliferation, restore endothelial function, reduce inflammation, and promote angiogenesis, largely through paracrine signaling [201,202]. Recent reviews also indicate that MSCs improve RV function in experimental PAH models, although the underlying mechanisms remain incompletely understood [202]. Human evidence includes a report of one child treated with UC-MSC-conditioned medium rather than MSC transplantation [203]. This uncontrolled observation does not establish clinical efficacy. The studies summarized below illustrate the main experimental and clinical findings.

PH can lead to RV hypertrophy and failure. Research conducted by Zhang et al. [204] focuses on the mechanism by which exosomes derived from UC-MSCs inhibit pulmonary vascular remodeling in a rat model of PH. An increase in the levels of BMP-4, BMP-9, and ID1 proteins was observed in samples obtained from the animals. It was concluded that the mechanism by which exosomes attenuate PH may be associated with the inhibition of NF-κB signaling, which leads to further activation of the BMP pathway. In contrast, Wang et al. [205] used a rat model of PH to analyze various BM-MSC treatment regimens, including early administration (1 day after injury), delayed administration on days 7 and 14 after injury, and multiple doses administered on days 1 and 11. MSC administration on day 1 significantly attenuated the progression of PAH by reducing RV systolic pressure and improving RV function. It also inhibited pulmonary small vessel remodeling. Repeated MSC administration did not yield better therapeutic effects than single-dose administration. Additionally, MSCs reduced the production of extracellular matrix proteins, which contributed to the inhibition of fibrosis in both in vitro models using pulmonary arterial adventitial fibroblasts and in vivo. A 2022 study reports the use of UC-MSCs in a 3-year-old girl with hereditary PH. The treatment lasted 6 months and improved hemodynamic parameters while reducing the levels of markers of vascular fibrosis and inflammation in the blood plasma. UC-MSCs were also used to perform single-cell RNA sequencing, which was compared to control human umbilical vein endothelial cells (HUVECs). Eight clusters with varying gene expression patterns among the cells were identified. The UC-MSC clusters were enriched in genes associated with regenerative pathways [203].

### 7.7. Heart Valve Diseases

Valvular heart disease is an increasingly important cause of cardiovascular morbidity, disability, and death, largely because populations are aging. In high-income countries, calcific aortic stenosis and degenerative mitral valve disease are the two most common forms of VHD, affecting approximately 9 million and 24 million people worldwide, respectively. In contrast, rheumatic heart disease remains the leading cause of mitral stenosis in low- and middle-income countries [206]. Global mortality from calcific aortic stenosis rose by 138% between 1990 and 2019, driven by aging populations in high-income countries and by the condition often being under-recognized and under-treated [206].

Interventional techniques such as transcatheter aortic valve implantation (TAVI) and transcatheter edge-to-edge mitral repair correct the mechanical consequences of valvular disease, while effective medical therapies targeting the underlying processes of primary valvular disease remain limited [207]. At the biological level, the pathophysiology of calcific aortic valve disease shares several fundamental features with atherosclerosis, including endothelial dysfunction, chronic low-grade inflammation, lipoprotein deposition, and progressive fibrosis and calcification of the valve leaflets. These changes are driven by pathological activation of valvular interstitial cells, a mechanism that resembles the processes underlying myocardial fibrosis. This mechanistic overlap suggests that calcific aortic valve disease may also be amenable to the anti-fibrotic and immunomodulatory effects of MSC therapy [206]. Early preclinical evidence is nonetheless emerging. In a murine model of aortic stenosis, MSC-derived exosomes enriched with nicotinamide phosphoribosyltransferase (NAMPT) reduced valvular thickening, calcification, and endothelial-to-mesenchymal transition (EndMT) [208], suggesting that exosome-based strategies could limit fibrocalcific remodeling [209]. Clinical evidence for MSC-based therapy in human valvular disease is still lacking.

MSC therapy in valvular disease is therefore at an early, exploratory stage. Progress in this field has been limited by three major challenges: the absence of dedicated clinical trials, the structural complexity of valve biology, and the availability of highly effective mechanical interventions. Nevertheless, the fibro-inflammatory nature of degenerative valve disease provides a rationale for MSC therapy as a potential adjunct capable of delaying disease progression before surgical or transcatheter intervention becomes necessary. Interest has also expanded toward valve tissue engineering, where MSCs are used both for scaffold recellularization and for their immunomodulatory properties.

Donor-related differences also need consideration. Cardiac-derived cells from a young patient with bicuspid aortic valve disease showed premature senescence [33]. Cells from one donor with endocarditis showed increased osteogenic differentiation and altered cardioprotective secretory activity [34]. These small studies support assessing the functional properties of each cell preparation. The canine study discussed below provides veterinary evidence, but does not establish efficacy in human valvular disease [210].

The research on the use of MSCs in heart valve diseases and heart valve tissue engineering is underway. Lanutti et al. [211] showed that Whorthon’s jelly-derived MSCs were able to decellularize porcine heart valves, and the interactions allowed the formation of neoendothelium, characterized by typical endothelial features. Later, Bozso et al. [212] showed that recellularization of xenogenic scaffolds with MSCs may be an effective approach to limit recipient immune response. They used BM-MSCs to decellularize bovine pericardium, which was then co-incubated with autologous whole blood. The results showed that decellularized bovine pericardium produced significantly lower levels of TNF-alpha and IL-1beta than decellularized bovine pericardium. Additionally, the levels were similar to those produced when human pericardium was exposed to whole blood. What is more, Kim and coworkers [213] recently demonstrated that using enzymes to remove xenoantigens from porcine pericardium scaffolds and decellularizing them with human AD-MSCs may be a promising strategy for producing biocompatible scaffolds via tissue engineering, for example, for the production of heart valves. The researchers demonstrated that this approach effectively increased cell infiltration, differentiation, and reendothelialization and reduced calcification. Attempts have also been made to use gonadal MSCs to treat early-stage myxomatous mitral valve disease (MMVD), the predominant heart disease in dogs. The study group received allogeneic MSCs intravenously at monthly intervals for five or more sessions. After one year of follow-up, echocardiographic deterioration was observed in the control group, while no differences were observed in the study group compared to baseline parameters. MSCs also had a beneficial effect on left atrial pressure, and their administration delayed disease progression [210].

## 8. Future Directions

Despite encouraging preclinical and early clinical data, MSC therapies are held back by the absence of standardized manufacturing and treatment protocols. Reaching consensus on cell source, dose, timing, delivery route, and patient selection will be essential because the current heterogeneity among clinical trials limits meaningful comparison. In our view, the near-term priorities are standardized manufacturing, potency assays linked to the proposed mechanism of action, and adequately powered trials of MSC products added to standard care. These steps address variability between cell preparations and uncertainty about clinical benefit. Preconditioning and biomaterial-assisted delivery should be advanced where they provide reproducible improvements in cell survival and activity. Cell-free extracellular vesicle therapies are a promising longer-term direction, but require standardized production, potency assessment, and clinical validation. Machine learning and multi-omics should initially support patient selection and biomarker development, with prospective validation before clinical use.

## 9. Conclusions

Among the cardiovascular disorders reviewed, IHD, acute MI, and HF with reduced ejection fraction are the most extensively studied applications of MSC-based therapy. Coronary artery diseases, including atherosclerosis and CMD, are an increasingly recognized part of ischemic disease, and cell-based endothelial repair offers a plausible mechanism to address them. Non-ischemic dilated cardiomyopathy is a clinically important secondary indication; systematic reviews and dedicated MSC trials support its feasibility. Current pharmacological, interventional, and device-based treatments improve symptoms and clinical outcomes, but do not replace lost cardiomyocytes. MSC-based therapies aim to support tissue repair through paracrine effects and should be evaluated as additions to standard care; their clinical efficacy remains uncertain. MSC-based approaches to myocarditis, PAH, and VHD remain at an early stage of research, with evidence coming mainly from preclinical studies and limited human observations. Despite differing etiologies and presentations, these disorders share several mechanisms—chronic inflammation, endothelial dysfunction, fibrosis, microvascular impairment, and cardiomyocyte loss—which is what makes them candidates for MSC-based therapy.

## Data Availability

No new data were created or analyzed in this study. Data sharing is not applicable to this article.

## Abbreviations

The following abbreviations are used in this manuscript:

2D	Two-dimensional
3D	Three-dimensional
6MWT	6-min walk test
AD-MSCs	Adipose-derived mesenchymal stromal cells
Akt	Protein kinase B
Akt1	AKT serine/threonine kinase 1
ANOCA	Angina with non-obstructive coronary arteries
ATP	Adenosine triphosphate
BAV	Bicuspid aortic valve
Bcl-2	B-cell lymphoma 2
BMCs	Bone marrow mononuclear cells
BM-MSCs	Bone marrow-derived mesenchymal stromal cells
BMP	Bone morphogenetic protein
BMP-4	Bone morphogenetic protein 4
BMP-9	Bone morphogenetic protein 9
BMPR2	Bone morphogenetic protein receptor type 2
CABG	Coronary artery bypass grafting
CAD	Coronary artery disease
CCR1	C-C chemokine receptor type 1
CD	Cluster of differentiation (including CD11b, CD14, CD19, CD34, CD45, CD73, CD79a, CD90, and CD105)
CI	Confidence interval
CMR	Cardiac magnetic resonance imaging
cMSCs	Cardiac-resident mesenchymal stromal cells
CMD	Coronary microvascular dysfunction
COVID-19	Coronavirus disease 2019
CRT	Cardiac resynchronization therapy
CVD	Cardiovascular disease
CXCR4	C-X-C chemokine receptor type 4
DFO	Deferoxamine
DMEM	Dulbecco’s Modified Eagle Medium
DMOG	Dimethyloxalylglycine
DMSO	Dimethyl sulfoxide
DNA	Deoxyribonucleic acid
DREAM-HF	Double-Blind Randomized Assessment of Clinical Events With Allogeneic Mesenchymal Precursor Cells in Heart Failure
EndMT	Endothelial-to-mesenchymal transition
EPPVDN	European Pediatric Pulmonary Vascular Disease Network
ESC	European Society of Cardiology
ESCaPE-CMD	Trial of autologous CD34+ cell therapy in coronary microvascular dysfunction
EVs	Extracellular vesicles
exo-miRs	Exosomal microRNAs
FBS	Fetal bovine serum
FGF-2	Fibroblast growth factor 2
FREEDOM	Placebo-controlled trial of CLBS16 in coronary microvascular dysfunction
GMP	Manufacturing compliant with good manufacturing practice
HF	Heart failure
HFrEF	Heart failure with reduced ejection fraction
HGF	Hepatocyte growth factor
HIF-1α	Hypoxia-inducible factor 1-alpha
HLA	Human leukocyte antigen
HLA-I	Human leukocyte antigen class I
HLA-II	Human leukocyte antigen class II
HLA-DR	Human leukocyte antigen-DR
HO-1	Heme oxygenase-1
hPL	Human platelet lysate
hMSCs	Human mesenchymal stromal cells
HUVECs	Human umbilical vein endothelial cells
IC	Intracoronary
ICD-11	International Classification of Diseases, 11th Revision
ICM	Ischemic cardiomyopathy
ID1	Inhibitor of DNA binding 1
IFN-γ	Interferon-gamma
IGF-1	Insulin-like growth factor 1
IHD	Ischemic heart disease
IL-1β	Interleukin-1 beta
IL-6	Interleukin-6
IL-10	Interleukin-10
IL-17	Interleukin-17
IM	Intramyocardial
INOCA	Ischemia with non-obstructive coronary arteries
IPC	Intrapericardial
I/R	Ischemia/reperfusion
IV	Intravenous
lncRNA	Long non-coding RNA
LVEDV	Left ventricular end-diastolic volume
LVEF	Left ventricular ejection fraction
LVESV	Left ventricular end-systolic volume
M2	M2 macrophage phenotype
MACE	Major adverse cardiovascular events
MI	Myocardial infarction
MINOCA	Myocardial infarction with non-obstructive coronary arteries
miRNA(s)	MicroRNA(s)
MLHFQ	Minnesota Living with Heart Failure Questionnaire
MMVD	Myxomatous mitral valve disease
MRI	Magnetic resonance imaging
mRNA	Messenger RNA
MSC(s)	Mesenchymal stromal cell(s)
mTOR	Mechanistic target of rapamycin
NAMPT	Nicotinamide phosphoribosyltransferase
NF-κB	Nuclear factor kappa B
NIDCM	Non-ischemic dilated cardiomyopathy
NLRP3	NLR family pyrin domain containing 3
NOGA	NOGA electromechanical mapping system
NT-proBNP	N-terminal pro-B-type natriuretic peptide
NYHA	New York Heart Association
PAB	Pulmonary artery banding
PAH	Pulmonary arterial hypertension
PBS	Phosphate-buffered saline
PCI	Percutaneous coronary intervention
PCL	Polycaprolactone
PET	Positron emission tomography
PGS	Poly(glycerol sebacate)
PH	Pulmonary hypertension
PI3K	Phosphoinositide 3-kinase
PLGA	Poly(lactic-co-glycolic acid)
POSEIDON-DCM	Percutaneous Stem Cell Injection Delivery Effects on Neomyogenesis in Dilated Cardiomyopathy
PRIME-HFrEF	Multi-intravenous infusion of umbilical cord mesenchymal stem cells in heart failure with reduced ejection fraction study
PTEN	Phosphatase and tensin homolog
PVD	Pulmonary vascular disease
ROS	Reactive oxygen species
RNA	Ribonucleic acid
RV	Right ventricle/right ventricular
RVESV	Right ventricular end-systolic volume
SARS-CoV-2	Severe acute respiratory syndrome coronavirus 2
SDF-1	Stromal cell-derived factor 1
SGLT2	Sodium-glucose cotransporter 2
SPIO	Superparamagnetic iron oxide
SVF	Stromal vascular fraction
TAVI	Transcatheter aortic valve implantation
TESI	Transendocardial stem cell injection
TGF-β	Transforming growth factor beta
TNF-α	Tumor necrosis factor alpha
UCB	Umbilical cord blood
UC-MSCs	Umbilical cord-derived mesenchymal stromal cells
VEGF	Vascular endothelial growth factor
VHD	Valvular heart disease
WHO	World Health Organization
WJ-MSCs	Wharton’s jelly-derived mesenchymal stromal cells
α-MEM	Alpha-Minimum Essential Medium
